# Biological mechanisms of dopamine D_2_-like receptor agonist therapy in diabetes

**DOI:** 10.3389/fendo.2025.1532414

**Published:** 2025-01-20

**Authors:** Zachary Freyberg, Ronald A. Codario

**Affiliations:** ^1^ Department of Psychiatry, University of Pittsburgh, Pittsburgh, PA, United States; ^2^ Department of Cell Biology, University of Pittsburgh, Pittsburgh, PA, United States; ^3^ Department of Medicine, Division of Endocrinology & Metabolism, University of Pittsburgh, Pittsburgh, PA, United States; ^4^ Department of Endocrinology, VA Pittsburgh Healthcare System, Pittsburgh, PA, United States

**Keywords:** dopamine, D_2_-like receptors, diabetes, dysglycemia, central nervous system, pancreas, adipocyte, T cells

## 1 Introduction

A recent case report by Sahota and colleagues has provided new insights into treatment of dysglycemia via dopamine (DA) receptor stimulation in the setting of autoimmune diabetes (1). Briefly, a patient with autoimmune diabetes was diagnosed with a pituitary prolactinoma, resulting in treatment with cabergoline, an agonist of DA D_2_-like receptors, alongside preexisting diabetes medications. Over time, the patient was switched to cabergoline monotherapy which reversed his insulin requirement. This led to significantly improved glycemic control and a revised diagnosis of latent autoimmune diabetes of adults (LADA). Ultimately, however, the patient was restarted on insulin therapy in the setting of progressively increased blood glucose.

Patients with LADA often achieve adequate glycemic control soon after the initiation of antihyperglycemic treatment, including non-insulin agents (2). Consistent with this, recent studies in LADA patients with non-insulin agents like dipeptidyl peptidase 4 inhibitors (e.g., saxagliptin), or glucagon‐like peptide 1 receptor agonists (e.g., dulaglutide) showed improved glycemic control for months and delayed progression to insulin requirement (2–5). Importantly, in contrast to the more commonly used drug classes above, this case represents the first description of DA receptor agonist monotherapy for autoimmune diabetes (1). These findings have raised important questions concerning the biological mechanisms by which D_2_-like receptor agonists can effectively treat dysglycemia, particularly in the setting of diabetes.

## 2 Discussion

### 2.1 CNS targets

D_2_-like receptor agonists such as cabergoline and bromocriptine have been used for decades to control CNS prolactinoma size and secretion given their expression of the DA D_2_ receptor (D2R) (6). There is much evidence that these agonists are associated with improved glycemic control (7). Moreover, bromocriptine was approved by the United States Food & Drug Administration as a novel treatment for dysglycemia in type 2 diabetes mellitus (T2DM) (8, 9). While mechanisms by which D_2_-like receptor agonists improve glycemic control have remained unclear, most attention has been devoted to these drugs’ actions on neuroendocrine targets within the central nervous system (CNS) (8).

**Figure 1:**
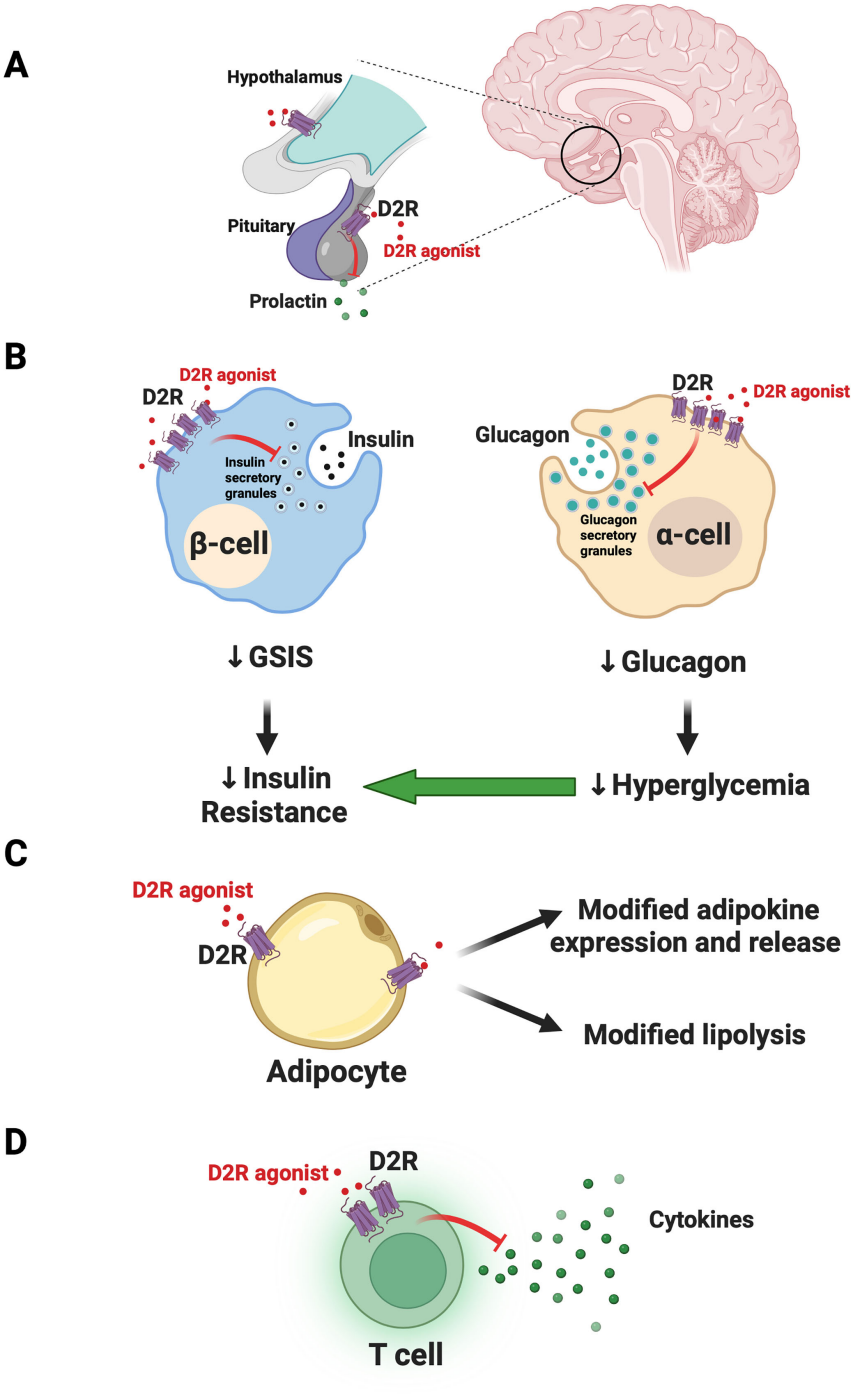
Model for joint actions of dopamine D_2_-like receptor agonist actions on CNS and peripheral targets to improve glycemic control. **(A)** In the CNS, dopamine D_2_-like receptor agonists like bromocriptine and cabergoline act on targets including the dopamine D_2_ receptor (D2R) in the pituitary to limit prolactin release. Targeting of additional hypothalamic targets may further modulate satiety and central metabolic circuitry to improve glycemic control. **(B)** Outside the CNS, in the endocrine pancreas, D_2_-like receptor agonists act on D2R expressed in beta-cells. The resulting inhibition of glucose-stimulated insulin secretion (GSIS) ultimately leads to therapeutic reductions in insulin resistance. In parallel, agonism of D2R in alpha-cells diminishes glucagon secretion to reduce hyperglycemia and further improve insulin sensitivity. **(C)** In adipose tissue, D_2_-like receptor agonists can act on D2R in adipocytes to modify release of adipokines and possibly lipolysis, improving insulin sensitivity. **(D)** D_2_-like receptor agonists also act on T cells in the endocrine pancreas to reduce cytokine release. This may reduce local inflammatory processes to improve islet function. Panel **(B)** was adapted from Aslanoglou et al. (2022) iScience 25 (2022) 104771. Created with BioRender.com.

Sahota et al. suggested that drug-induced reduction of pathological prolactin levels led to the patient’s metabolic improvements (1). CNS D2R agonism via cabergoline therapy could therefore modify prolactin-induced disruptions in lipid and glucose metabolism in insulin-responsive tissues including adipose tissue and skeletal muscle (1, 10, 11). These prolactin reductions also likely contributed to improved testosterone levels, which in turn reversed the patient’s hypogonadism. This is consistent with evidence showing that testosterone restoration contributes to significant weight loss as well as improved insulin resistance and overall glycemic control (12, 13). Cabergoline-induced normalization of prolactin may therefore lead to restored total and free testosterone levels to improve glycemic control via a wide range of mechanisms including via reductions in inflammation and weight gain – factors that further drive insulin resistance (11, 14). Moreover, D2R is also expressed in the hypothalamus and is implicated in centrally-mediated metabolic regulation, including through control over satiety (15, 16). Therefore, it is possible that D2R agonists may improve glycemic control via these CNS pathways, in addition to its actions in the pituitary (**Figure 1A**).

Though CNS DA receptor agonism was proposed by Sahota et al. as a primary driver of improved glycemic control (1), additional factors likely play key roles. A leading determinant of improved glucose control is the “honeymoon” effect where patients present with temporary remission after symptomatic onset. The honeymoon period in LADA typically lasts weeks to months and may reflect reduced stress on remaining islet beta-cells (17–19). Body weight loss similarly improves glycemic control, which in turn lowers cell stress to help preserve beta-cell function (e.g., insulin synthesis and release) (20).

### 2.2 Endocrine pancreas

In addition to CNS targets, we posit that the ability of D_2_-like receptor agonists to effectively treat dysglycemia in diabetes is at least in part via their actions on metabolically relevant peripheral targets including the endocrine pancreas. We and others demonstrated that pancreatic islet cells express D_2_-like receptors (21–26). Moreover, alpha-cells and beta-cells produce their own DA which signals locally via D_2_-like receptors as an autocrine/paracrine negative modulator of insulin and glucagon secretion (21, 22, 26, 27). More recently, we found that bromocriptine acts directly on peripheral D2R to inhibit islet insulin and glucagon secretion (28). It is possible that D_2_-like receptor agonist inhibition of glucose-stimulated insulin secretion (GSIS) therefore leads to “beta-cell rest.” Lowering excessive insulin release may reduce cytotoxic beta-cell stress and re-sensitize insulin-resistant tissues like skeletal muscle, adipose tissue, and liver to improve dysglycemia (26). Interestingly, besides DA receptors, beta-cells also express inhibitory adrenergic receptors including alpha_2A_ adrenergic receptors which can similarly be stimulated by local DA or D_2_-like receptor agonists like bromocriptine (22, 28, 29). This results in further inhibition of GSIS (22, 28, 29). Likewise, diminishing alpha-cell glucagon secretion via D2R agonism may concurrently lower hyperglycemia and improve both insulin resistance and overall glycemic control (26) (**Figure 1B**).

### 2.3 Adipose tissue

D_2_-like receptors are expressed in adipose tissue (30, 31). Increasing evidence suggests that dopaminergic signaling in adipocytes modulates expression of adipokines including leptin (32–34). Consistent with this, recent work showed that D2R expression was upregulated in human subcutaneous adipose tissue in response to hyperglycemia and T2DM (34). The DA D_4_ receptor (DRD4), another D_2_-like receptor, was also upregulated in adipose tissue of patients with prediabetes (35). Moreover, bromocriptine treatment inhibited lipolysis in response to beta-adrenergic receptor stimulation, suggesting that D_2_-like receptor agonists may be acting directly on adipocytes to modify their function (34). Despite this, the same study reported that physiological concentrations of DA did not modify either adipocyte glucose uptake or lipolysis (34). This raises the possibility that D_2_-like receptor agonists achieve their therapeutic effects via actions at additional non-dopaminergic adipocyte receptors. It is also possible that at least some of the therapeutic effects of D_2_-like receptor agonists on peripheral insulin resistance are due to pleotropic, combined actions at multiple peripheral sites which include adipocytes, but which also include other sites such as liver. Indeed, earlier work demonstrated that bromocriptine treatment led to remodeling of adipose tissue with increases in fasting insulin signaling in brown adipose tissue (35). In parallel, bromocriptine may also act on liver (e.g., diminished liver triglyceride content) (35). Ultimately, more work is clearly needed to investigate direct and indirect therapeutic actions of D_2_-like receptor agonists on adipocyte function (**Figure 1C**).

### 2.4 Skeletal muscle

In addition to adipose tissue, skeletal muscle also plays a key role in maintaining adequate peripheral insulin sensitivity and optimal glucose control. However, effects of D_2_-like receptor agonists on skeletal muscle are mixed. Limited preclinical evidence in rodents showed that bromocriptine increased phosphorylation of skeletal muscle AMP-activated protein kinase (AMPK), an energy-sensing enzyme and therapeutic target in diabetes (36, 37). In contrast, other preclinical and clinical studies showed no significant effects of bromocriptine on skeletal muscle, including on insulin sensitivity (35, 38). Nevertheless, in the case of the patient described by Sahota and colleagues (1), irrespective of potential direct actions of a D_2_-like receptor agonist on skeletal muscle, drug-induced restoration of serum levels of testosterone may lead to improved skeletal muscle mass and strength and improve insulin sensitivity (12, 39).

### 2.5 T cells

Immune T cells that have infiltrated pancreatic islets represent another possible peripheral therapeutic target for D_2_-like receptor agonists. Immune cells express D_2_-like receptors and stimulation of these receptors can decrease cytokine secretion, potentially suppressing activated actions of islet T cells (40). We therefore posit that resulting decreases in islet inflammation can improve islet function and glycemic control (**Figure 1D**).

### 2.6 Tandem CNS and peripheral dopaminergic actions

We recently found that D_2_-like receptor agonists required access to both CNS and peripheral targets to treat dysglycemia. Importantly, restricting access to one compartment or the other eliminated the therapeutic efficacy of the agonist drugs in reducing dysglycemia (41). Overall, we conclude that tandem actions of D_2_-like receptor agonists on CNS and peripheral targets offer a novel mechanism for dysglycemia treatment of autoimmune diabetes and T2DM.
